# Factors associated with severe malaria among children below ten years in Mutasa and Nyanga districts, Zimbabwe, 2014-2015

**DOI:** 10.11604/pamj.2017.27.23.10957

**Published:** 2017-05-10

**Authors:** Faith Mutsigiri-Murewanhema, Patron Trish Mafaune, Gerald Shambira, Tsitsi Juru, Donewell Bangure, More Mungati, Notion Tafara Gombe, Mufuta Tshimanga

**Affiliations:** 1 Department of Community Medicine, University of Zimbabwe, Harare, Zimbabwe; 2 Ministry of Health and Child Care, Manicaland Province, Zimbabwe

**Keywords:** Severe malaria, risk factors, Mutasa, Nyanga, Zimbabwe

## Abstract

**Introduction:**

Severe malaria is a rare life threatening illness. Only a small proportion of patients with clinical malaria progress to this medical emergency. On reviewing 61 malaria death investigation forms submitted to the provincial office in 2014, 22(36%) were children below ten years who succumbed to severe malaria. Mutasa and Nyanga Districts reported 73% of these deaths. This study was conducted to determine factors associated with severe malaria so as to come up with evidence based interventions to prevent severe malaria and associated mortality.

**Methods:**

A 1:2 unmatched case control study was conducted. A case was defined as a child 10 years and below, who was admitted at Hauna (Mutasa) or Nyanga District Hospitals between September 2014 and May 2015 with a primary diagnosis of severe malaria. Controls were children of similar age with uncomplicated malaria. Permission to conduct the study was sought and granted by the Medical Research Council of Zimbabwe (Approval number B/874), Joint Research Ethics Committee, Health Studies Office and the Manicaland Directorate Institutional Review Board. Written informed consent was sought from all caregivers of enrolled children. Interviewer administered questionnaires were used to ascertain exposures.

**Results:**

A total of 52 cases and 104 controls were enrolled into the study. The median age of cases was 4 years (Q_1_=3, Q_3_=9) and 6 years for controls (Q_1_=3, Q_3_=8). The Case Fatality Rate among cases was 28.8%. The independent risk factors for severe malaria were; distance >10km to the nearest health facility [Adjusted Odds Ratio (aOR)=14.35, 95% CI=1.30, 158.81], duration of symptoms before seeking medical care >2 days [aOR=9.03, 95% CI=2.21, 36.93], having comorbidities [aOR=5.38, 95% CI=1.90, 15.19], staying in a house under construction [aOR=4.51, 95%CI=1.80, 11.32] and duration of illness before receiving antimalarial medicines >24 hours [aOR=3.82, 95% CI=1.44, 10.12]. Owning at least one ITN in the household [aOR=0.32, 95% CI=0.11, 0.95] and having a mother as a caregiver [aOR=0.23, 95% CI=0.09, 0.76] were independently protective of severe malaria. Being undernourished [Odds Ratio (OR)=10.13, 95% CI=1.04, 98.49] and being female [OR=0.27, 95% CI=0.08, 0.96] were associated with mortality owing to severe malaria.

**Conclusion:**

Factors associated with severe malaria and mortality owing to severe malaria identified in this study are consistent with other studies. Caregiver healthcare seeking behaviours, patient related factors and health system related factors are important determinants of severe malaria among children. There is need for regular health education campaigns emphasizing on malaria prevention, signs and symptoms and benefits of seeking medical care immediately for sick children.

## Introduction

Malaria is among the world's commonest and life threatening tropical diseases. Malaria is caused by Plasmodium parasites which are transmitted through the female Anopheles mosquito's bite which occurs mainly between dusk and dawn [1]. In humans, malaria is caused by 4 parasites namely; Plasmodium falciparum, Plasmodium vivax, Plasmodium malariae and Plasmodium ovale. P. falciparum and P. vivax are the commonest [1], however, P. falciparum remains the single most important threat to public health at a global scale since it is the most deadly. It accounts for more than 90% of the world's malaria mortality [2]. Malaria is endemic in most tropical regions and about 3.4 billion people worldwide are exposed to malaria annually and 1.2 billion are at high risk [1]. Although preventable and curable, malaria causes significant morbidity and mortality especially in regions with limited resources [3]. An estimated 300-500 million people suffer from malaria every year and 1.5-2.7 million deaths occur [4, 5]. Sub-Saharan Africa is the most affected region [3] contributing over 80% of global malaria deaths [6]. Although half of the world's population is at risk of malaria, and whilst anyone living or visiting a malaria endemic area may be at risk, vulnerability is higher in certain groups, particularly pregnant women and children. Malaria is a leading cause of death among children less than 5 years, who represent 77% of all global malaria deaths [7, 8]. In Africa a child dies every minute from malaria [1]. Children are mostly affected because their immune systems are not yet fully developed to fight severe forms of disease [7, 8]. Pregnant women have a reduced immunity hence they have increased risk of infection with malaria, severe disease and ultimately increased risk of death [9, 10].

Malaria remains one of the top three causes of child mortality in sub-Saharan Africa, including Zimbabwe. It is a major public health problem in Zimbabwe with almost half of the population at risk. Approximately 1 in 12 children in Zimbabwe die before their 5th birthday due to malaria [11], this translates to 84 deaths per 1000 live births [12]. Plasmodium falciparum accounts for 97% of cases seen in health facilities in Zimbabwe [13]. If not treated within 24 hours, P. falciparum malaria can progress into fatal severe illness [1]. Early malaria diagnosis and treatment reduces disease, prevents deaths and can contribute to the reduction of malaria transmission. Mild cases of malaria are easily treatable with complete recovery using relatively inexpensive and widely available first line drugs. However, treatment is complicated and expensive for severe malaria. Mortality is also higher among children who present with severe malaria than among those with mild/ uncomplicated disease. On reviewing 61 malaria death investigation forms submitted to the provincial office in 2014, it was noted that 22(36%) were children below ten years who succumbed to severe malaria and of these, 14(64%) were under-fives. Mutasa and Nyanga Districts contributed the majority of these deaths with 16 of the 22 deaths (73%) reported from these two districts. Malaria morbidity and mortality remains high despite scaling up of the implementation of malaria control interventions by the national malaria control programme in the country. A major question of concern is why only a small proportion of infected individuals progress to severe and life-threatening illness. The objective of this study was to assess the factors associated with severe malaria among children below ten years.

## Methods

An unmatched 1:2 case control study was conducted in Mutasa and Nyanga Districts, Manicaland Province, Zimbabwe. A case control study design was chosen because severe malaria is a rare disease and study participants were recruited on the basis of the disease status. The study design enabled determination of multiple exposures for a single outcome at once.

**Study Setting**: The study was conducted in health facilities and communities in Mutasa and Nyanga Districts of Manicaland Province.

**Study Population**: The study population were children under-ten years diagnosed of simple malaria and severe malaria between September 2014 and May 2015, their parents/caregivers and medical records.

**Working definitions**: For the purposes of this study, cases and controls were defined as follows;

**Case**: A child 10 years and below who was admitted at Hauna (Mutasa) or Nyanga District Hospitals in Manicaland Province between September 2014 and May 2015 with a primary diagnosis of severe malaria (defined by the presence of microscopy detected P. falciparum parasites together with at least one of the following features: convulsions before/during admission, severe anaemia (haemoglobin <5g/dl), cerebral malaria (impaired consciousness), prostration and/or respiratory distress (deep breathing and/or chest in drawing).

**Control**: A child 10 years and below who had uncomplicated malaria, defined by the presence of a fever (axillary temperature >37.5^0^C) and a positive Rapid Diagnostic Test result for malaria parasites, but without any features of severe malaria between September 2014 and May 2015, and also residing in the same area with a case.

**Inclusion criteria**: Child below 10 years with a primary diagnosis of WHO-defined severe malaria and was admitted at Hauna or Nyanga District hospitals. (Cases); exclusion of other common known causes of any of the manifestations of severe malaria e.g meningitis. (Cases); child below 10 years with simple malaria diagnosed at a health facility which serves the areas where cases came from.(Controls); a patient who was resident in Mutasa or Nyanga District as confirmed by the patient's usual address recorded in the hospital records. During follow up of study participants to their homesteads, their parents/guardians also confirmed the residence status; informed consent was obtained from the child's caregiver for inclusion in the study.

**Exclusion criteria**: A patient who was visiting Mutasa or Nyanga District as confirmed by the patient's usual address recorded in the hospital records; a child whose caretaker denied to consent for participation in the study.

**Sample size determination**: Sample size was calculated using the Stat Calc function of Epi info version 7, using a confidence level of 95% and power of 80%. Based on a study by Imani et al “Human immunodeficiency virus infection and cerebral malaria in children in Uganda: a case-control study,” assumptions were that age under-five years (0-59 months) was a significant risk factor for severe malaria (Odds Ratio of 2.47). The prevalence of exposure among controls was 37.2% [14]. Based on this information, to conduct a 1:2 unmatched case control study the required minimum sample size was 65 cases and 130 controls.

### Sampling procedure

**Sampling of cases**: A sampling frame of all children who had severe malaria and were hospitalized at Hauna and Nyanga District hospitals in Manicaland Province between September 2014 and May 2015 was developed. All the 52 children who had severe malaria and satisfied the inclusion criteria after reviewing medical records were enrolled into the study. The children were traced to their homes using addresses on the patients' admission notes and their caregivers were interviewed.

**Sampling of controls**: Following recruitment of each case, two controls who resided in the same area with a case were recruited. Systematic sampling was used to randomly select controls from children who were attended for simple malaria at a health facility that serves the area where a case resided from September 2014 to May 2015. The clinic's daily attendance register was the sampling frame for controls. Selection of controls was limited to those who resided in the same area with a case. Children who were chosen to participate in the study were then traced to their homes using addresses in the admission and outpatient registers and their caregivers were interviewed.

**Permission to conduct study and ethical considerations**: Permission to conduct the study was sought from the Manicaland Provincial Medical Directorate Institutional Review Board, District Medical Officers for Mutasa and Nyanga and the Health Studies Office. Clearance to conduct the study was obtained from the Joint Research Ethics Committee (JREC) and the Medical Research Council of Zimbabwe (Approval number B/874). The aim of the study was explained to all the parents/guardians of potential study participants and all were assured that they were free to withdraw from the study at any time and no penalty would be imposed on them or their child if they decided to do so. Informed written consent was sought from caregivers of enrolled children. Confidentiality was assured and maintained throughout the study. Caregivers of children selected to participate in the study answered the questions. In the event that the child was deceased the caregivers were told to be prepared for emotional disturbance before the interview and those who required professional counselling were referred.

**Data collection**: Pretested interviewer administered questionnaires were used to collect data on socio-demographic characteristics, household related factors, patient related factors, health system related factors and caregiver healthcare seeking behavioural factors. Patients' medical records at the health facility were also reviewed to assess eligibility for participation in the study. Parents/caregivers of cases and controls were interviewed in the community.

**Data analysis**: Data were analysed using Epi info version 3.5.3 to calculate frequencies, proportions and means. The same statistical package was used to calculate measures of association and their 95% confidence intervals. Stepwise forward logistic regression was done for all variables that were significantly associated with severe malaria at the p=0.25 level on bivariate analysis to determine the independent factors associated with severe malaria.

## Results

A total of 52 cases and 104 controls were enrolled into the study. The majority of both cases and controls were female; 55.8% and 52.9% respectively. The highest proportion of controls, (58.7%), were aged above five years old, whilst the majority of cases, 51.9%, were aged 5 and below. The median age of cases was 4 years (Q1=3; Q3=9) and 6 years (Q1=3; Q3=8) for controls. Fifty-one (98.1%) cases and 100(96.2%) controls resided in rural areas. The majority of the cases, 35(67.3%) presented with convulsions. The least common manifestation of severe malaria was severe anaemia, only 1(1.9%) case presented with it. The Case Fatality Rate among cases was 28.8%. Table 1 summarizes the factors associated with severe malaria among children below ten years in Mutasa and Nyanga Districts. Caregiver factors associated with severe malaria among children were; having at least secondary education [Odds ratio (OR) =0.73, 95% CI=0.37, 1.43], being the mother of the child [OR=0.41 95% CI=0.18, 0.91] and female caregiver [OR=0.36, 95% CI=0.07,1.66]. Environmental factors that were significantly associated with severe malaria were; staying in house under construction [OR=3.89, 95% CI=1.927.88>7.88], staying in a house with open eaves or poorly covered windows [OR=2.09, 95% CI=1.06, 4.12], stagnant water within 10 meters from household [2.08, 95% CI=1.01, 4.28] and having received IRS 12 months preceding child's illness [OR=0.39, 95% CI=0-20, 0.77]. Household related factors that were significantly protective of severe malaria were; sleeping under mosquito net every night [ OR=0.33, 95% CI=0.16, 0.70] and owning at least one ITN in the household [OR=0.23, 95% CI=0.10, 0.51]. Patient related factors associated with severe malaria were; HIV positive status [OR=4.69 95% CI=1.73, 12.60], having comorbidities [OR=3.44, 95% CI=1.59, 7.44], undernutrition [OR=3.40, 95% CI=31.24, 9.34] and history of malaria illness [ OR=0.48, 95% CI=0.24, 0.96]. Health system factors that were significantly associated with severe malaria among children were; distance between home and nearest health facility >10km [OR=10.96, 95% CI=1.25, 96.41], delayed diagnosis [OR=5.24, 95% CI=1.29, 21.18] and having received antimalarial medicines at initial visit to a health facility [OR=0.19, 95% CI=0.05, 0.77].

**Table 1:** Factors associated with severe malaria among children below ten years, Mutasa and Nyanga districts, Zimbabwe, 2014-2015

Variable	Cases n (%)	Controls n (%)	OR (95% CI)	p-value
At least secondary education	28(53.8)	64(61.5)	0.73 (0.37-1.43)	0.36
Being mother of the child	36(69.2)	88(84.6)	0.41 (0.18-0.91)	0.02
Female caregiver	48(92.3)	101(97.1)	0.36 (0.07-1.66)	0.17
Staying in a house under construction	35(67.3)	36(34.6)	3.89 (1.92-7.88)	<0.01
Staying in a house with open eaves or poorly covered windows	31(59.6)	43(41.3)	2.09 (1.06-4.12)	0.03
Stagnant water within 10 meters from household	20(38.5)	24(23.1)	2.08 (1.01-4.28)	0.04
Received IRS 12 months preceding child’s illness	22(42.3)	68(65.4)	0.39 (0.20-0.77)	0.01
Own at least 1 ITN in household	32(61.5)	91(87.5)	0.23 (0.10-0.51)	<0.01
Child sleeps/slept under mosquito net every night	13(25.0)	52(50.0)	0.33 (0.16-0.70)	<0.01
Positive HIV status	16(53.3)	10(19.6)	4.69 (1.73-12.69)	<0.01
Having comorbidities	20(38.5)	16(15.4)	3.44 (1.59-7.44)	<0.01
Under nutrition	17(63.0)	14(33.3)	3.40 (1.24-9.34)	0.02
History of malaria illness	20(38.5)	59(56.7)	0.48 (0.24-0.96)	0.04
Distance between home and the nearest health facility >10km	5(9.6)	1(1.0)	10.96(1.25-96.41)	<0.01
Delayed diagnosis	7(13.5)	3(2.9)	5.24 (1.29-21.18)	0.01
Child managed by a VHW	17(32.7)	25(24.0)	1.53 (0.74-3.20)	0.35
Received antimalarial at initial visit to a health facility	45(86.5)	101(97.1)	0.19 (0.05-0.77)	0.01
Duration of symptoms before seeking medical care >2 days	19(36.5)	4(3.8)	14.30(4.57-45.36)	<0.01
Duration of illness before child received antimalarial >24hrs	38(73.1)	34(32.7)	5.59 (2.67-11.68)	<0.01
Administered medication at home before seeking medical care	23(44.2)	19(18.3)	3.55 (1.69-7.43)	<0.01
First action taken when child got sick was seeking medical care	28(53.8)	84(80.8)	0.28 (0.13-0.58)	<0.01

Children who were managed by a VHW were 1.53 times more likely to have severe malaria than those who were not. However this finding was not statistically significant [95% CI=0.74, 3.20]. Caregiver healthcare seeking behaviors that were significantly associated with severe malaria were; duration of child's symptoms before seeking medical care >2 days [OR=14.30, 95% CI=4.57, 45.36], duration of illness before child received antimalarial >24hrs [OR=5.59, 95% CI=2.67,<11.68],medication at home before seeking medical care (OR=3.55> administering medication at home before seeking medical care (OR=3.55, 95% CI=1.69, 7.43] and first action taken when child got sick was seeking medical care [OR=0.28, 95% CI=0.13, 0.58]. Of the 52 children with severe malaria, 15 died and 37 recovered. A sub-analysis was done to determine factors associated with mortality due to severe malaria. Table 2 summarizes the factors that were associated with mortality due to severe malaria among children below ten years in Mutasa and Nyanga Districts. The patient related factors that were associated with mortality were; undernutrition [OR=10.13, 95% CI=1.04-98.49], age <5 years [OR=2.35, 95% CI=0.67, 8.24] and HIV positive status [OR=1.22, 95% CI=0.22, 6.73]. Caregiver healthcare seeking behavioural factors that were associated with mortality due to severe malaria were; duration of child's symptoms before seeking medical care >2 days [OR=2.70, 95% CI=0.79, 9.29] and first action taken was seeking medical care [OR=0.45, 95% CI=0.13, 1.55] though not statistically significant. The independent risk factors for severe malaria were; distance >10km to the nearest health facility [Adjusted Odds Ratio (aOR)=14.35, 95% CI=1.30, 158.81], duration of symptoms before seeking medical care >2 days [aOR=9.03, 95% CI=2.21, 36.93], having comorbidities [OR=5.38, 95% CI=1.90, 15.19], staying in a house under construction [aOR=4.51, 1.80, 11.32] and duration of illness before receiving antimalarial medicines >24 hours [aOR=3.82, 95% CI=1.44,10.12]. Owning at least one ITN in the household [95% CI=0.11, 0.95] and having a mother as a caregiver [aOR=0.23, 95% CI=0.09, 0.76] were independent protective factors for severe malaria **Table 3**.

**Table 2:** Factors associated with mortality due to severe malaria among children below ten years, Mutasa and Nyanga districts, 2014-2015

Variable	Category	Died n (%)	Recovered n (%)	OR(95%CI)	p-value
Child undernourished	Yes	9(90.0)	8(47.1)	10.13(1.04-98.49)	0.03
	No	1(10.0)	9(52.9)		
Delayed Diagnosis	Yes	3(20.0)	4(10.8)	2.75(0.49-15.53)	0.24
	No	12(80.0)	33(89.2)		
Duration of child’s symptoms before seeking medical care >2 days	Yes	8(53.3)	11(29.7)	2.70(0.79-9.29)	0.11
	No	7(46.7)	26(70.3)		
Age <5 years	Yes	10(66.7)	17(45.9)	2.35(0.67-8.24)	0.18
	No	5(33.3)	20(54.1)		
HIV Status	Positive	4(57.1)	12(52.2)	1.22(0.22-6.73)	0.82
	Negative	3(42.9)	11(47.8)		
Having comorbidities	Yes	6(40.0)	14(37.8)	1.10(0.32-3.74)	0.89
	No	9(60.0)	23(62.2)		
First action taken was seeking medical care	Yes	6(40.0)	22(59.5)	0.45(0.13-1.55)	0.20
	No	9(60.0)	15(40.5)		
Sex	Female	5(33.3)	24(64.9)	0.27(0.08-0.96)	0.04
	Male	10(66.7)	13(35.1)		

**Table 3:** Independent factors associated with severe malaria among children below ten years, Mutasa and Nyanga districts, 2014-2015

Variable	AOR	95%CI	Coefficient	p-value
Distance between home and nearest health facility >10km	14.35	1.30-158.81	2.66	0.03
Duration of child’s symptoms before seeking medical care >2 days	9.03	2.21-36.93	2.20	<0.01
Having comorbidities	5.38	1.90-15.19	1.68	<0.01
Staying in a house under construction	4.51	1.80-11.32	1.51	<0.01
Duration of illness before child received antimalarial >24hrs	3.82	1.44-10.12	1.34	<0.01
Own at least 1 ITN	0.32	0.11-0.95	-1.14	0.04
Having mother as a caregiver	0.23	0.09-0.76	-1.36	0.01
Constant	+	+	-1.15	0.12

## Discussion

Distance of more than 10km to the nearest health facility was an independent risk factor for severe malaria. Time spend travelling to a health facility and associated transport costs can influence the decision to seek treatment early for malaria and therefore result in delayed diagnosis and treatment as caregivers opt for initial treatment at home. In a study by Malik et al in 2006 in Sudan, the choice of treatment for sick children among caregivers was highly dependent on accessibility and availability of health facilities [15]. Most people in rural areas live further away from health facilities. To address the issue of long distances between communities and health facilities which may result in delayed diagnosis and treatment, Zimbabwe introduced community case management of malaria by Village Health Workers (VHWs) in 2012. VHWs were introduced to bring essential health services closer to the people, hence they should have adequate malaria commodities at all times if their existence is to make a difference. Duration of symptoms before seeking medical care for the sick child of more than 2 days was an independent risk factor for severe malaria. Duration of illness >24 hours before receiving antimalarial medicines and delayed diagnosis were also significantly associated with severe malaria in children. This was consistent with findings in a study by Byakika-Kibwika et al (2009) in Uganda [16]. Malaria is an emergency because of its capability to progress to severe, fatal illness if not treated appropriately and promptly [17]. Quite a number of children die because of malaria within 24-72 hours of onset of symptoms [18]. Timeous diagnosis and treatment is therefore crucial to prevent progression of disease to severe form and ultimately lower mortality. Presumptive treatment with antimalarial medicines of all fevers in children who live in malaria endemic areas is the main strategy for reducing malaria related child morbidity and mortality [19].

Having comorbidities was found to be independently associated with severe malaria. Similar findings were reported in several studies [20–22]. Co-infection with plasmodium parasites and HIV infection is of importance. HIV infection and was found to be a risk factor for severe malaria. Infection with HIV suppresses the immune system hence increases the individual's susceptibility to many other infections. HIV infection also decreases response to antimalarial medicines, thereby increasing the burden of the diseases [14]. Similar findings were also reported in studies in Kenya, Uganda and South Africa were HIV infection was found to be associated with severe malaria in children [14, 23–25]. Staying in a house under construction was an independent risk factor for severe malaria in this study. A partially complete house without windows or a roof or with other openings may facilitate frequent and repeated exposure to parasite infected mosquitoes because mosquitoes gain access to the inside of the house through these openings thereby exposing the habitants to infective bites. Complete and good house construction is a barrier to malaria transmission because it limits access of mosquitoes to the household. Siri et al (2010) reported similar findings in Kenya, however the results were not significant on multivariate analysis [26]. Owning at least one ITN in the household was an independent protective factor against severe malaria. Sleeping under a mosquito net every night was also significantly protective of severe malaria. Bed net use offers personal protection from getting mosquito bites. Widespread ITN use has been seen to reduce malaria morbidity and mortality in Kenya and Nigeria [27, 28]. Having a mother as a caregiver was independently protective of severe malaria. Mothers tend to seek care immediately for their sick children and they pay particular attention to their children's needs. It therefore makes it easier for a mother to notice that their child is not feeling well. In rural settings most mothers are not employed, they spend most of their time at home with the children. This creates a bond between mother and child, hence children are most likely to tell their mother if they are not feeling well and the mother takes prompt action thereby decreasing chances of disease progression to severe form. Since child care is normally done by mothers, we suspect that having a caregiver who was not the mother may mean that the child is orphaned and this may lead to late identification of signs of disease and late presentation to health facilities for medical care.

Under-nutrition was a significant risk factor for severe malaria and associated with mortality in children. In a study by Caulfield et al in 2004, improved nutritional status was seen to reduce malaria related deaths because it lessens the severity of malaria episodes [29]. Undernutrition reduces functionality of all systems of the body. This has great consequences especially in young children [29]. Underweight children have increased susceptibility to malaria through impairment in the function of the immune system. Undernourished children may be incapable to mount appropriate immune response to parasites causing malaria because of reduced T-lymphocytes, impairment of antibody formulation and atrophy of the thymus and other lymphocyte tissues [30]. History of malaria illness prior to the recent illness was significantly associated with reduced odds of severe malaria. Similarly, in a study by Phillips history of malaria was seen to be protective of developing severe disease, probably through acquired immunity [31]. Related findings suggest acquiring some form of protection following at least one infection [32]. The CFR was high in this study. This can be attributed to the fact that the majority of the cases, were from rural areas. It has been noted that about 50% of those who develop severe malaria especially in remote areas die. This is because health services are faraway and are not well equipped to manage complications caused by the diseases [33, 34]. The CFR was higher (28.8%) compared to that in a study in Ghana by Mockenhaupt et al in 2004 (11.2%) [35]. In this study the high CFR can be attributed to convulsions which was the commonest manifestation of severe malaria among cases in this study. The CFR of severe malaria is dependent on the predominant manifestations that also have implications on the treatment [35].

**Limitations**: The required minimum sample size was not achieved because most children who were admitted with a diagnosis of severe malaria did not suit the WHO case definition. The study sample came from a population who sought treatment at the health facilities, therefore the findings from this study cannot be generalized regarding the health care seeking behaviours of every caregiver. There was a possibility of recall bias because some cases and controls were recruited months after their illness. The study may also have been prone to under-reporting and over-reporting especially of healthcare seeking behaviours of caregivers because it was partly based on self-reporting. Retrospective record review may risk misclassification of cases and controls

## Conclusion

Severe malaria was characterized by a high proportion of convulsions and the CFR was high in this study. The factors associated with severe malaria and mortality due to severe malaria was identified. There is need for prompt treatment with antimalarial medicines of all high risk patients like undernourished children and those infected with HIV to avoid further progression of disease to severe form. There is need for stronger program linkages e.g Malaria, HIV and Nutrition. There is need for training of health workers on manifestations of severe malaria to avoid over diagnosis. Scaling up community health education and promotion campaigns emphasizing on consistent and correct use of preventive strategies like ITNs is essential. A Prospective study is necessary to address some of the limitations of this study.

### What is known about this topic

- Malaria due to plasmodium falciparum is the third leading cause of mortality among children in Zimbabwe;
- Approximately 1 in 12 children in Zimbabwe die before their 5th birthday due to malaria;
- Mortality and complications due to malaria can be prevented by early seeking of appropriate health care.

### What this study adds

- This study corroborates findings from previous studies that the following are risk factors for severe malaria in children; HIV coinfection, undernutrition and delay in seeking medical care;
- There is need to integrate malaria, HIV and Nutrition programs in order to minimize complications associated with comorbidities of these diseases;
- Having a mother as the primary caregiver is protective of severe malaria.

## Competing interests

The author declare no competing interest.
